# Patients with allergic rhinitis and allergic asthma share the same pattern of eosinophil and neutrophil degranulation after allergen challenge

**DOI:** 10.1186/1476-7961-9-3

**Published:** 2011-01-21

**Authors:** Mary Kämpe, Ingrid Stolt, Maria Lampinen, Christer Janson, Gunnemar Stålenheim, Marie Carlson

**Affiliations:** 1 Department of Medical Sciences, Respiratory Medicine and Allergology, Uppsala University, Uppsala, Sweden; 2 Asthma Research Centre, Uppsala University, Uppsala, Sweden; 3 Department of Medical Sciences, Gastroenterology Research Group, Uppsala University, Uppsala, Sweden

## Abstract

**Background:**

Patients with allergic rhinitis and allergic asthma demonstrate comparable local and systemic eosinophil inflammation, and yet they present with different clinical pictures. Less is even known about the contribution of neutrophil inflammation in allergic diseases. The aim of the study was to examine the propensity and selectivity of granule release from primed systemic eosinophils and neutrophils in allergic rhinitis and allergic asthma after seasonal and experimental allergen exposure. We hypothesize that the dissimilar clinical manifestations are due to diverse eosinophil and neutrophil degranulation.

**Methods:**

Nine birch pollen allergic patients with rhinitis, eight with asthma and four controls were studied during pollen season and after nasal and bronchial allergen challenge. Eosinophils and neutrophils were incubated in vitro with assay buffer and opsonized Sephadex particles for spontaneous and C3b-induced granule protein release. The released amount of eosinophil cationic protein (ECP), eosinophil peroxidase (EPO) and myeloperoxidase (MPO) was measured by specific radioimmunoassay.

**Results:**

C3b-induced degranulation resulted in increased release of ECP and MPO from primed blood eosinophils and neutrophils in both allergic rhinitis and allergic asthma during pollen season and after both nasal and bronchial challenge (p-values 0.008 to 0.043). After bronchial challenge, the ECP release was significantly higher in the rhinitic group compared to the asthmatic group [19.8 vs. 13.2%, (p = 0.010)]. The propensity for EPO release was weak in all challenge models but followed the same pattern in both allergic groups.

**Conclusions:**

Systemically activated eosinophils and neutrophils have similar patterns of degranulation after allergen exposure in allergic rhinitis and allergic asthma. The released amount of ECP, EPO and MPO was similar in all allergen challenge models in both allergic groups. Our results indicate that other mechanisms than the magnitude of eosinophil and neutrophil inflammation or the degranulation pattern of the inflammatory cells determines whether or not an allergic patient develops asthma.

## Introduction

Allergic diseases, such as allergic asthma, allergic rhinitis and atopic dermatitis are characterised by an increased number of eosinophil granulocytes in the circulating blood and degranulation in the target tissue is considered the major pathogenic event [1]. The eosinophil is a multifunctional leukocyte playing a central role in Th_2 _mediated allergic diseases [2], parasitic killing and tissue repair [1]. Recent studies have also pointed out eosinophil involvement in modulating both innate and adaptive immune responses [3]. The primed eosinophil rapidly secretes four preformed, highly cytotoxic, cationic granule proteins at the site of inflammation: eosinophil cationic protein (ECP), eosinophil peroxidase (EPO), eosinophil derived neurotoxin (EDN)/former eosinophil protein X (EPX) and major basic protein (MBP) beside chemokines, cytokines and growth factors [1,3]. In addition to regulated exocytosis and cytolysis [4], the eosinophils release their granule proteins through a process of piecemeal degranulation by transport vesicles allowing selective release of the eosinophilic granule proteins [5,6].

Jatakanon *et al *reported more than a decade ago that neutrophils have an important role in chronic severe asthma [7], and neutrophil inflammation of the airways is today considered relevant to the pathogenesis of the more severe forms of the disease [8,9]. However, in a novel study, neutrophilia was observed in induced sputum in children with non-atopic asthma [10], but the role of neutrophils in allergic rhinitis and mild asthma is uncertain and under debate. It has been speculated that neutrophils are taking part in both the initiation and resolution of even mild asthma attacks [8].

The neutrophils house two major granule populations, primary (azurophil) and secondary (specific) granules, formed during the maturation process. The primary granules contain mainly myeloperoxidase (MPO), several proteases and the antibiotic defensin peptides, all released in a potentially active state [11]. The specific granules store latent pro-forms of mainly metalloproteases, activated by the azurophilic proteases first after the degranulation [11]. The highly cytotoxic myeloperoxidase from the primary granules has been used as a marker of the neutrophil activity [12].

It has been known for long that binding of eosinophils and neutrophils to a surface by complement receptors induces a strong signal for degranulation, involving the receptor for complement factor 3 (C3b receptor) [13,14]. Using serum-opsonised Sephadex particles *in vitro *in experimental settings [15,16] enhances this C3b-induced degranulation of the eosinophils in allergy as well as in infections [17,18]. Previous studies have reported increased propensity of granule release *in vitro *from primed eosinophils and neutrophils in allergic asthma compared to controls after Sephadex stimulation, both during pollen season as well as out of season [19,20]. This data indicates priming of both types of granulocytes in allergic asthmatics.

The link between the upper and lower airways is well-established [21]. Many studies have reported both blood eosinophilia and local eosinophilia in nasal lavage as well as in induced sputum both during pollen season and after local allergen challenge in the nose and bronchi respectively [22-24]. The question remains why patients with allergic asthma and allergic rhinitis demonstrate more or less the same degree of systemic eosinophil inflammation both during pollen season and after nasal and bronchial challenge and yet they present with different clinical pictures. The hypothesis of the present study was that the dissimilar clinical manifestations of asthmatic and rhinitic patients are due to differences in selective eosinophil and neutrophil degranulation. The primary aim of the study was thus to study differences in allergic rhinitis and allergic asthma with regard to the degranulation pattern of allergen primed eosinophils and neutrophils. A secondary aim was to investigate if there is a differential and selective granule release from primed eosinophils and neutrophils in the two allergic groups depending on the allergen challenge model.

## Materials and methods

### Patients

Seventeen birch pollen allergic patients were selected for the study, all diagnosed with seasonal allergic rhinitis or allergic asthma by a lung physician and allergologist at the allergy out-patient clinic at Uppsala University Hospital All patients were skin prick test positive to birch pollen and none of the patients had symptoms or were on any regular treatment outside birch pollen season. Eight patients had a diagnosis of allergic seasonal asthma, having respiratory symptoms (wheeze and dyspnea) and denying nasal symptoms during birch pollen season, and thus were categorised as having asthma as the predominant symptom. Nine patients were diagnosed with allergic rhinitis, having eye and nose symptoms and denying respiratory symptoms, and consequently categorised as having rhinitis as the predominant symptom. Topical steroids were not allowed during pollen season or outside season, and none of the patients were on any regular medication during season. None of the patients had smoked for the past ten years. Forced expiratory volume in one second (FEV_1_) out of season was more than 75% of predicted and FEV_1_/forced vital capacity (FVC) more than 70% in all patients (Table 1).

**Table 1:** Demographic data of the control group and patients with allergic rhinitis and allergic asthma (mean and range).

	Control group	Allergic rhinitis	Allergic asthma	p-value
	n = 5	(n = 9)	(n = 8)	(AR/AA)***
**Gender **(male/female)	2/3	8/1	3/8	0.36

**Age**	38 (27-58)	43 (24 - 66)	41 (19 - 56)	0.85

Ex-smoker (>10 yr)	0	2	1	0.79

**SPT birch **(in mm^2^)	0	47.1 (20 - 88)	43.6 (26 - 64)	1.0

**IgE for birch**, Class 0-5	0	3.1 (2 - 4)	3.4 (2 - 5)	0.43

FEV_1 _(L)	3.59(3.02-3.95)	4.0 (2.4 - 4.9)	3.5 (2.6 - 4.0)	0.12

**FEV**_**1 **_**% **of predicted	105 (88-125)	102 (75 - 139)	97 (83 - 108)	0.44

**PEFR **(L/min)	571 (348-854)	615 (415 - 826)	504 (347 - 652)	0.18

**PEFR % **of predicted	117 (84-169)	113 (82 - 140)	101 (73 - 133)	0.25

**FEV**_**1**_**-decrease **in % *	1.1 (-0.5-6.2)	0.42 (-5.6 - 5.8)	6.81 (1.55 - 16.4)	*0.02*

**PD**_**20 **_(birch) (SQE)**	-	34 500 (3850 - 150 000)	3 700 (2450 - 7700)	*0.04*

**Morning PEFR **(L/min) diary during pollen season	-	575 (550 - 620)	475 (433 - 551)	*0.02*

**Evening PEFR **(L/min) diary during pollen season	-	610 (555 - 630)	478 (449 - 551)	*0.005*

* After inhalation of hypertonic 4.5% saline solution at baseline.

** After bronchial allergen challenge (median and range).

*** AR = allergic rhinitis and AA = allergic asthma.

### Control group

The control group consisted of five healthy, non-atopic, never smoking subjects, having allergic symptoms neither outside nor during the birch pollen season. They were skin prick test negative to all nine standard allergens, had no serum IgE antibodies, and had normal lung function with an FEV_1 _>80% of predicted. The control group only completed investigations during the pollen season (Table 1).

### Study design

The study included altogether five visits to our out-patient clinic: inclusion, baseline, during birch pollen season and after bronchial and nasal allergen challenge respectively. The season visit was made two to three weeks after the airborne pollen counts had reached 4 000 grains/m^3^, pollen grains counted by the Palynological Laboratory, Swedish Museum of Natural History, Stockholm, Sweden [23]. The study was performed during the birch pollen seasons in 2000 and 2002; the season 2001 was excluded due to low pollen counts. After inclusion patients were investigated consecutively, thus all patients were studied pre-season and during season in the same year. Bronchial and nasal allergen challenges were performed during a four week period in January and February the following year. The subjects were told to avoid short-acting bronchodilators and anti-histamines for 24 hours before the visits and nasal decongestants for four hours before the visits. When pollen counts reached 4 000 grains/m^3 ^the subjects were told to start recording their morning and evening PEFR in a diary. The design of the present study has been described in detail in previous reports [23,24].

### Skin prick tests

Skin prick tests were performed with nine standard aeroallergen extracts (birch, timothy, mugwort, cat dander, dog dander, horse dander, *Dermatophagoides pteronyssinus, Cladosporium herbarum *and *Alternaria *using Soluprick SQ ALK (Hørsholm, Denmark). The results were read after 15 minutes, measuring the largest diameter of the wheal and its perpendicular diameter, and the product was expressed in mm^2^. Skin reactions were considered positive when larger than 9 mm^2^.

### Spirometry

Lung function tests were performed with a Vitalograph-Compact spirometer (Vitalograph Ltd., Buckingham, England). FEV_1_, FVC, FEV_1_/FVC% and PEFR were recorded. The reference values were those from European Community for Coal and Steel [25]. Spirometry was performed before and after the hypertonic saline inhalation. The magnitude of the FEV_1 _decrease after the hypertonic saline inhalation was used as a marker of bronchial responsiveness [23].

### Nasal challenge test

The experimental nasal challenge test was performed by instillation in the same nostril of 0.3 mL diluent followed by birch pollen extract (Aquagen^® ^SQ, ALK-Abelló, Hørsholm, Denmark) every 15 minutes in three steps: 1 000 SQ-U/mL, 10 000 SQ-U/mL and 100 000 SQ-U/mL. The symptom score was estimated; if pronounced local symptoms and sneezing occurred, the challenge test was stopped. The response to the allergen provocation was categorized into four groups: no response or response to one or more of the three allergen doses. Blood samples and nasal lavage were taken 18 hr (±1 hr) after the challenge test was completed.

### Bronchial allergen challenge test

The experimental bronchial challenge test was performed using a DeVilbiss-40 nebulizer (particle size 0.5 to 5.5 μm, output 0.175 ± 0.3 mL/min, mean ± SD) (Devillbiss Co, Somerset, PA) [26]. Bronchial challenge with birch pollen extract (Aquagen^® ^SQ, ALK-Abelló, Hørsholm, Denmark) was performed in three steps with the doses 1 000 SQE, 10 000 SQ-U and 100 000 SQ-U, starting with inhalation of a diluent. The response to the allergen provocation was calculated as the cumulative dose that caused at least 20% decrease in FEV_1 _(allergen provocation dose, PD_20_). The challenge test was stopped if FEV_1 _decreased by 20%. Blood samples were taken after 18 hr (±1 hr).

### Isolation of granulocytes

Isolation was performed on heparinized blood. The mononuclear leukocytes were separated by percoll gradient centrifugation [27]. The erythrocytes were lysed by ice-cold, sterile water and then washed. The granulocyte mixture obtained by this procedure had a purity of 99.8% ± 0.2% (SD). The cell viability after this procedure was 99.0-99.5%, determined by staining with Trypan blue.

### Inflammatory cell counts

Four ml of EDTA blood was collected for routine laboratory tests of eosinophil and neutrophil counts (Cell-Dyn 4000, Abbott Laboratories, Abbot Park, Illinois, USA) at the accredited laboratory at the Department of Clinical Chemistry, Uppsala University Hospital. Differential cell counts were obtained using a cytospin preparation (Cytospin, Shandon, Southern Instruments, Sewickley, PA, USA), stained with May-Grünewald-Giemsa and examined under light microscope.

### Radioimmunoassays (RIA) of ECP, EPO and MPO and RadioAllergoSorbent Test (RAST)

The released amounts of ECP and MPO from the eosinophils and neutrophils, respectively, were assayed by means of specific RIA (Pharmacia Diagnostics AB, Uppsala, Sweden) and EPO with ImmunoCAP FEIA (Pharmacia Diagnostics AB, Uppsala, Sweden). Specific IgE was determined with RAST (Pharmacia Diagnostics AB, Uppsala, Sweden) at the Department of Clinical Immunology, Uppsala University Hospital (normal <0.35 kU/L).

### Calculations of released amounts of ECP, EPO and MPO

The amounts of released ECP, EPO and MPO were expressed as percent of total cellular content, calculated from a standard curve of serial dilutions of respective cell extracts. Results were calculated by regression analysis.

### Measurement of eosinophil and neutrophil degranulation

The assay for C3b-mediated granule release by Sephadex-particles, was performed according to Winquist *et al *[13] with some minor modifications as previously described [20]. The final concentration of granulocytes in the assay was 1.0 × 10^9^/L. The cells were pre-incubated for 10 min with assay buffer. Incubation was then performed at 37°C for 0 and 20 min with assay buffer for spontaneous granule release or washed, with serum-treated Sephadex G-15 particles for stimulated granule release (83.5 g/L) [GE Healthcare (formerly Amersham Biosciences) NJ, USA] for stimulated release. Hanks' solution supplemented with 0.74 mM Ca^2+ ^and 0.1% human serum albumin (HSA) was used as assay buffer. All incubations were made in duplicate. For measurement of total cell content of granule proteins; 300 mL of granulocytes (3.0 × 10^9^/L) was mixed with 1.5 mL of 0.5% N-acetyl-N,N,N-trimethylammonium bromide in 0.15 mM NaCl and then incubated for 1 hr at room temperature followed by centrifugation at 600 *g *for 10 min at 4°C. The volume of 1.5 mL of supernatant was removed and stored for later measurement of granule proteins. The released amounts of granule proteins were expressed as % of total cell content.

#### Ethical approval

The study was performed with the approval of the ethics committee at the Medical Faculty at Uppsala University and informed consent was obtained from each subject.

#### Statistical evaluation

The Kruskal-Wallis, ANOVA and Mann-Whitney U test were used to evaluate statistical differences between patient groups. For paired analyses, we used Friedman's ANOVA and Wilcoxon's matched pairs test. Correlations were investigated with Spearman's test (rho). A p-value of < 0.05 was considered significant. All the calculations were performed using the statistical software package Statistica (Statsoft Inc, Tulsa, Oklahoma, USA).

## Results

### Clinical characteristics

No significant differences at baseline concerning gender, age, smoking, allergy variables and lung function were recorded between patients with allergic rhinitis and allergic asthma. However, patients with allergic asthma were more responsive as measured by FEV_1_-decline to inhalation of hypertonic 4.5% saline solution at baseline, had a greater decrease in both morning and evening PEFR during pollen season and also had a greater responsiveness expressed as allergen PD_20 _for birch after bronchial challenge [23,24], (Table 1).

### Spontaneous degranulation (0 to 20 min) of ECP, EPO and MPO in assay buffer

#### Pollen season

There were no significant increases in degranulation of ECP, EPO or MPO in patients with allergic rhinitis, allergic asthma (Table 2, 3 and 4) or in the control group.

**Table 2:** ECP release from eosinophils spontaneously and after C3b-stimulation (at 0 and 20 min) in allergic rhinitis, allergic asthma and the control group during pollen season and after nasal and bronchial challenge.

	Spontaneous degranulation of ECP*		p-value	Stimulated degranulation of ECP*		p-value
	(median, range)		increase 0-20 min	(median, range)		increase 0-20 min
	**0 min**	**20 min**		**0 min**	**20 min**	

**Pollen season**						

*Allergic rhinitis*	1.78	2.04	0.12	2.42	17.9	*0.01*
	(0.24 - 2.88)	(0.38 - 3.17)		(0.66 - 3.79)	(9.21 - 28.6)	

*Allergic asthma*	1.48	1.70	0.40	2.05	14.0	*0.02*
	(0.99 - 7.10)	(0.8 - 7.32)		(0.76 - 8.97)	(7.13 - 45.1)	

**Nasal challenge**						

*Allergic rhinitis*	1.23	2.02	*0.04*	1.24	19.5	*0.04*
	(0.17 - 2.03)	(0.78 - 2.46)		(0.31 - 2.7)	(14.7 - 23.5)	

*Allergic asthma*	1.78	2.12	*0.02*	1.72	14.9	*0.02*
	(0.97 - 3.07)	(1.14 - 3.92)		(0.88 - 2.49)	(8.06 - 26.8)	

**Bronchial challenge**						

*Allergic rhinitis*	1.50	2.08	0.12	2.45	19.8	*0.03*
	(0.99 - 2.38)	(1.36 - 3.16)		(1.08 - 3.21)	(15.5 - 24.3)	

*Allergic asthma*	1.66	1.68	0.4	1.38	13.2	*0.02*
	(0.12 - 1.92)	(0.6 - 2.11)		(0.08 - 2.02)	(9.70 - 17.1)	

* Release of ECP in % of total cell content

#### Nasal challenge

The release of ECP increased significantly in both patients with allergic rhinitis and allergic asthma (Table 2). A significant increase of MPO was also demonstrated in patients with allergic asthma (Table 4). For EPO no significant increase in degranulation was presented in either allergic group (Table 3).

**Table 3:** EPO release from neutrophils spontaneously and after C3b-stimulation (at 0 and 20 min) in allergic rhinitis and allergic asthma during pollen season and after nasal and bronchial challenge.

	Spontanous degranulation of EPO		Stimulated degranulation of EPO		p-value	p-value
	**0 min**	**20 min**	**0 min**	**20 min**		

**Pollen season**						

*Rhinitics*	0.42	0.37	0.45	2.05	0.18	0.02*
	(0.12 - 0.63)	(0.13 - 0.61)	(0.35 - 0.92)	(1.08 - 3.0)		

*Asthmatics*	0.32	0.30	0.39	1.58 -	0.59	0.11
	(0.17 - 0.52)	(0.08 - 0.43)	(0.38 - 0.74)	(1.52 - 3.46)		

**Nasal challenge**						

*Rhinitics*	0.50	0.49	0.61	1.4	0.9	0.07
	(0.38 - 0.62)	(0.36 - 0.62)	(0.51 - 0.71)	(1.2 - 1.6)		

*Asthmatics*	0.49	0.44	0.54	1.86	0.07	0.07
	(0.45 - 0.69)	(0.42 - 0.46)	(0.48 - 0.74)	(1.54 - 3.22)		

**Bronchial challenge**						

*Rhinitics*	0.28	0.32	0.55	1.99	0.7	0.04*
	(0.21 - 0.46)	(0.16 - 0.40)	(0.26 - 0.93)	(1.71 - 2.17)		

*Astmatics*	0.44	0.41	0.52	1.84	0.14	0.07
	(0.31 - 0.62)	(0.26 - 0.56)	(0.35 - 0.64)	(1.04 - 2.04)		

* Release of EPO in % of total cell content

**Table 4:** MPO release from neutrophils spontaneously and after C3b-stimulation (at 0 and 20 min) in allergic rhinitis and allergic asthma during pollen season and after nasal and bronchial challenge.

	Spontaneous degranulation of MPO*		p-value	Stimulated degranulation of MPO*		p-value
	(median, range)		increase 0-20 min	(median, range)		increase 0-20 min
	**0 min**	**20 min**		**0 min**	**20 min**	

**Pollen season**						

*Allergic rhinitis*	2.12	2.45	0.81	2.84	15.9	*0.008*
	(1.18 - 6.86)	(1.67 - 5.64)		(0.93 - 7.07)	(10.6 - 29.2)	

*Allergic asthma*	2.25	2.50	0.74	3.05	17.6	*0.018*
	(0.28 - 5.05)	(0.95 - 5.5)		(0.37 - 5.20)	(7.48 - 24.0)	

**Nasal challenge**						

*Allergic rhinitis*	2.61	3.16	0.68	2.93	14.9	*0.043*
	(1.42 - 5.64)	(1.68 - 4.1)		(1.63 - 5.55)	(12.7 - 21.7)	

*Allergic asthma*	2.21	3.31	*0.018*	2.35	18.4	*0.018*
	(1.18 - 5.62)	(2.08 - 7.40)		(1.35 - 5.22)	(12.6 - 25.4)	

**Bronchial challenge**						

*Allergic rhinitis*	2.42	3.43	0.075	3.88	21.6	*0.028*
	(2.17 - 2.92)	(2.24 - 4.93)		(2.14 - 6.00)	(16.8 - 27.3)	

*Allergic asthma*	1.50	1.91	*0.018*	1.58	16.8	*0.018*
	(0.84 - 2.3)	(1.36 - 2.48)		(0.71 - 2.96)	(13.1 - 23.3)	

*release of MPO in % of total cell content

#### Bronchial challenge

The spontaneous release of MPO significantly increased in the asthmatic group, but not in patients with allergic rhinitis (Table 4). For ECP and EPO no significant increases in degranulation could be recorded (Table 2 and 3).

### C3b-stimulated degranulation (0 to 20 min) of ECP, EPO and MPO

#### Pollen season

**Figure 1:**
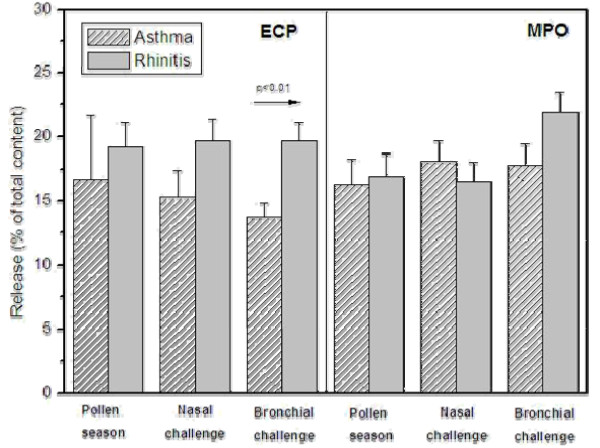
**C3b-induced degranulation of eosinophil cationic protein (ECP) and myeloperoxidas (MPO) (at 20 min), in patients with allergic asthma and allergic rhinitis, during pollen season and after nasal and bronchial challenge, respectively**.

A significant increase of ECP and MPO could be recorded in both patients with allergic rhinitis and allergic asthma (Table 2 and 4, Figure 1). However, EPO release increased significantly only in patients with allergic rhinitis (Table 3). In the control group no increases in ECP, EPO or MPO were observed.

#### Nasal challenge

ECP increased significantly in both patients with allergic rhinitis and allergic asthma (Table 2, Figure 1). A significant increase of release of MPO was also seen in the two allergic groups (Table 4, Figure 1). No significant increase in EPO degranulation was detected in either the rhinitic or asthmatic patients (Table 3).

#### Bronchial challenge

Both ECP and MPO increased significantly in both allergic groups (Table 2 and 4, Figure 1). The increase in EPO degranulation was statistically significant only in patients with allergic rhinitis but not in the asthmatic group (Table 3).

### Degranulation (0 to 20 min) in allergic rhinitis compared to allergic asthma

No significant differences in the degree of spontaneous degranulation of ECP, EPO or MPO could be recorded between patients with allergic rhinitis and allergic asthma in either allergen challenge model. After *in vitro *stimulation with Sephadex particles, the increased degranulation of ECP was significantly higher in the rhinitic than the asthmatic group (p = 0.010), (Figure 1). There was a similar tendency for stimulated MPO release in allergic rhinitis but this was not significant.

### Relationship between the released amount of granule proteins, clinical data and systemic inflammation

No correlation between degranulation and lung function (measured as FEV_1 _or PEFR) or blood parameters (B-eosinophils, S-ECP or S-HNL) could be observed.

## Discussion

The main finding of our study was that all three allergen challenge models could prime both eosinophils *and *neutrophils to an increased propensity of selective degranulation after stimulation *in vitro *by opsonised Sephadex particles. Remarkably, there was no significant difference in the degranulation response between patients with allergic rhinitis and allergic asthma except for a significantly greater release of ECP in the rhinitic patients after bronchial allergen challenge (p = 0.010). The three provocation models also primed the granulocytes for degranulation on a comparable level even though the systemic inflammation was more pronounced during long-term pollen exposure compared to single-dose allergen challenge [24]. This again highlights the close relationship between the upper and lower airways, but it also raises new questions about the cellular nature of inflammation in atopy.

The eosinophil granulocytes account for 1-2% of the circulating white blood cells but they are primarily tissue-residing cells in the hematopoietic organs as well as in the airways, the gastrointestinal tract and the skin. The physiological function of the eosinophils is not completely understood, but they are known to be involved in the innate immune response against parasitic infections, tissue repair and recently it has been discovered that they also have the ability to modulate immune responses [3]. The activation of the eosinophils is strictly regulated as an inappropriate activation would be harmful to the subject and in healthy conditions the eosinophils are inactivated with a high threshold for release of their granule proteins [28]. However, after stimulation the activated eosinophils are primed for extensive degranulation in the different target organs, expressing high-affinity IgE-receptors (Fcε-receptors), Fcγ-receptors and complement receptors [3,19]. *In vitro *studies have demonstrated selective release of the individual granule proteins [19], and interestingly, different eosinophilic diseases are characterized by a marked heterogeneity in degranulation levels [29]. Previous studies suggested that the priming-degree of the blood eosinophils is related to the degranulation status of the tissue-residing eosinophils and so corresponds to the activity of the eosinophilic disease [30].

Previous analyses of the study population have shown that the asthmatic group was more responsive to inhalation of hypertonic saline [23], had more pronounced lung function impairment during the pollen season [23], and was more responsive to allergen PD_20 _after bronchial challenge than the rhinitic group [24]. Despite these differences, both patient groups showed a similar degree of eosinophil inflammation both locally and systemically during pollen season as well as after both nasal and bronchial challenge [23,24]. Our hypothesis was therefore that differences in degranulation patterns contribute to the outcome of different clinical manifestations between the allergic groups. However, the results in this study did not support this hypothesis.

We found that both in patients with allergic rhinitis and allergic asthma, the released amount of ECP after C3b-induced stimulation was in the same range during pollen season as after both nasal and bronchial challenge. Surprisingly, we also recorded the same pattern for stimulated MPO release in both patient groups. Our interpretation is that seasonal exposure as well as nasal and bronchial allergen challenge can activate, prime, eosinophils and neutrophils more or less to the same degree. The tendency that patients with allergic rhinitis and allergic asthma display the same pattern of degranulation of ECP and MPO is in line with previous observations from our group where we demonstrated an increased propensity of ECP and EPX/EDN secretion during pollen season in patients with allergic asthma [19]. In that study, however, we only recorded a slight tendency of increase for MPO [19]. This could partly be explained by the fact that the granulocytes in the present study were pre-incubated for 10 min with assay buffer, which was not the case in our previous paper. The results indicate that priming of granulocytes is also applicable for patients with allergic rhinitis and follows the same pattern as for patients with allergic asthma. The observation of neutrophil activation in both allergic groups is in contrast to data that other groups have reported in which mild and moderate asthmatics did not display any neutrophil inflammation [8].

Stimulated EPO degranulation tended to increase in the rhinitic patients compared to the asthmatics both during pollen season and after nasal and bronchial challenge. However, there was only a minor absolute increase in EPO release even after C3b-induced stimulation in both allergic patient groups. This discrepancy between the release of ECP and EPO is an interesting finding considering that EPO is regarded to be the most specific eosinophil granule protein [31]. Our data is in line with previous reports from both our and other groups where it has been observed that EPO is more difficult to mobilize than ECP [29,30,32]. This difference could be explained by selective granule release in response to different stimuli for degranulation [30], as EPO is a potent enzyme and perhaps plays a more important role in the innate defence against parasites and not primarily in allergy.

We were intrigued by the observation that the rhinitic patients showed a higher release of ECP and MPO after bronchial allergen challenge than the asthmatic patients. One interpretation could be that the granulocytes of the patients with allergic asthma are easier to prime and activate, particularly after bronchial allergen challenge, and therefore already have released their granule proteins in response to the allergen exposure. This hypothesis is supported by a slightly higher amount of ECP per eosinophil cell prior to the C3b-induced granule release after bronchial challenge in the rhinitic patients compared to the asthmatics (mean 3.01 vs. 2.73 μg ECP/B-eos 10^6^). This is in accordance with results from other groups that have observed hypodense blood eosinophils after allergen exposure, implicating degranulation in response to allergen challenge [5]. On the other hand, Malm-Erjefält *et al *evaluated patients with allergic asthma, allergic rhinitis and atopic dermatitis with regard to intracellular EPO by transmission electron microscopy, demonstrating no degranulation of the eosinophils in circulating blood. The degranulation status was, however, based on the cell content of EPO [33]. This is in line with our results and also with previous studies where it has been observed that EPO is more difficult to mobilize from the primed blood eosinophils [20,30,32].

Eosinophils have been considered as major effector cells in the pathogenesis of asthma, but the role of the neutrophils is less understood in the allergic airway inflammation except in more severe forms of chronic asthma [34]. Histologically, the asthmatic lung is characterized by an eosinophil-rich inflammation and by a variety of chronic changes including remodelling and deposition of extracellular matrix components [35,36]. Interestingly, Phipps *et al *recently showed that even in mild atopic asthma acute allergen-induced remodelling could occur early [37], and in another study neutrophils were prominently elevated in asthma exacerbations [38]. The novel finding of neutrophils in induced sputum of non-atopic asthmatic children [10] also points in the direction of the neutrophils playing an important role, not just in severe chronic stages of the disease, but also in mild disease. Additionally, the recent advances using anti-IL-5 therapy indicate involvement of other inflammatory cells than just the eosinophils, as the bronchial hyperresponsiveness is not affected by anti-IL-5 therapy despite depletion of the eosinophils from circulation by this treatment [39]. Altogether, this implies that there might not be a clear-cut difference between mild and severe asthma with regard to the neutrophil involvement, and thus eosinophilic and neutrophilic asthma might not be mutually exclusive subtypes of asthma.

The strength of our study is the simultaneous evaluation of the priming status of the eosinophils and neutrophils in blood after both long-term natural allergen exposure during pollen season and a single high-dose allergen challenge in the nose and bronchi in both allergic rhinitics and allergic asthmatic patients concurrently. One drawback of this study is the relative small number of subjects in each allergic group which limited the opportunity to find differences between the two allergic groups, but the results imply that blood granulocytes of both allergic rhinitis and allergic asthma are more or less equally primed for chemotaxis and degranulation in their target tissue. However, there are many questions to be resolved and further investigations are needed in order to study the degranulation process at the site of action.

## Conclusion

In conclusion, patients with allergic rhinitis and allergic asthma display similar patterns of eosinophil and neutrophil propensity for degranulation when exposed to allergen. However, there is a tendency to increased release in the rhinitic patients, but this only significant for ECP release after bronchial challenge. Our results indicate that other mechanisms than the magnitude of inflammation and degranulation patterns of the inflammatory cells determine whether or not an allergic patient with rhinitis develops asthma.

## Competing interests

The authors declare that they have no competing interests.

## Authors' contributions

MK, MC, CJ and GS designed the study and were responsible for analyzing and interpreting the results as well as critically revising the manuscript. IS carried out the assays and degranulation measurements. ML was involved in drafting the manuscript and the figures. All authors have contributied in reading an improving the manuscript.
